# 恶性胸膜间皮瘤45例临床分析

**DOI:** 10.3779/j.issn.1009-3419.2012.02.06

**Published:** 2012-02-20

**Authors:** 洁 陈, 微 赵, 星辰 刘, 庆 田, 震 杨, 良安 陈

**Affiliations:** 100853 北京，解放军总医院呼吸内科 Department of Respiratory Medicine, Chinese PLA General Hospital, Beijing 100853, China

**Keywords:** 恶性间皮瘤, 胸膜, 诊断, 治疗, Malignant mesothelioma, Pleural, Diagnosis, Treatment

## Abstract

**背景与目的:**

恶性胸膜间皮瘤是一种罕见疾病，其发病率在逐年上升，早期诊断和治疗非常困难。本文旨在探讨恶性胸膜间皮瘤的临床特点、诊断及治疗，为临床提供参考。

**方法:**

回顾性分析解放军总医院1997年1月-2010年12月收治的45例恶性胸膜间皮瘤患者的临床资料。

**结果:**

恶性胸膜间皮瘤患者的主要临床症状为胸痛（53.33%）、胸闷气促（48.89%）和咳嗽（37.78%）；CT表现主要为胸膜增厚（71.11%）、胸腔积液（60%）和肺部阴影（40%）；胸水以渗出液为主，有核细胞数明显增多，以单核细胞的增多为主，乳酸脱氢酶明显增高；大部分患者临床分期为Ⅲ期和Ⅳ期；确诊的方式主要是胸腔镜，病理类型以上皮型多见，且常易被误诊为结核性胸膜炎。早期患者以手术治疗为主，而晚期患者以化疗为主，病理类型为上皮型的疾病控制率高于肉瘤型。

**结论:**

恶性胸膜间皮瘤误诊率较高，其临床症状无特异性，胸部CT可提供诊断依据，组织病理学检查结合免疫组化才能确诊，治疗方式包括化疗、手术、放疗和支持治疗，普遍疗效欠佳。

## Abstract (en)

**Background and objective:**

Malignant pleural mesothelioma (MPM) is a rare tumor with increasing incidence, and its early diagnoses and early treatments are difficult. The aim of the current study is to investigate the clinical features, diagnoses, and treatments of MPM, and provide reference for clinical use.

**Methods:**

A total of 45 MPM patients admitted in the Chinese PLA General Hospital from January 1997 to December 2010 and their clinical records were analyzed retrospectively.

**Results:**

The major clinical symptoms of the patients included: chest pain (53.33%), chest distress (48.89%) and cough (37.78%). The CT manifestations of MPM included: pleural thickening (71.11%), pleural effusion (60%) and lung shadow (40%). The pleural fluid was exudative. The number of nucleated cells in the fluid increased, most of which were mononuclear cells. Moreover, the lactic acid dehydrogenase (LDH) levels in the culture medium significantly increased. The most common clinical stage of MPM patients was either stage of Ⅲ or Ⅳ. Thoracoscopy was one of the main diagnosis methods, and epithelium cell type was the main pathologic type. In addition, MPM was easily misdiagnosed as tuberculous pleurisy. Patients with an early stage of MPM were mainly cured through surgery, whereas chemotherapy was applied for the patients with advance stage. The disease control rate of the patients with epithelial type was higher than that of the patients with fibrosarcoma type.

**Conclusion:**

The rate of MPM misdiagnosis is high because most of the patients have untypical clinical symptoms. However, CT scan can provide an important basis for the diagnosis of MPM. The accurate diagnoses were based on pathological examinations and immunohistochemistry. The methods of treatment include chemotherapy, surgical operation, radiotherapy and supportive treatment. However, these treatments are still insufficient and require further studies.

恶性胸膜间皮瘤（malignant pleural mesothelioma, MPM）是原发于胸膜间皮组织的一种少见的进展性胸部恶性肿瘤，其发病与石棉接触密切相关。此病恶性程度高，早期诊断困难，目前尚无有效治疗措施，病死率高，预后差。随着工业的发展，特别是石棉的广泛应用，该病的发病率有明显增加的趋势，因此愈来愈引起人们的关注^[1]^。本文选取解放军总医院1997年1月-2010年12月确诊的MPM患者45例进行分析。

## 1 资料与方法

收集解放军总医院1997年1月-2010年12月经组织病理学确诊的45例MPM患者的临床资料，包括病史、临床症状、胸部影像学、胸水常规和生化指标、病理组织及细胞学检查、治疗方式及疗效等方面进行回顾性分析。使用SPSS 16.0统计学软件进行数据处理。

## 2 结果

### 2.1 一般临床资料

患者平均年龄为52岁，范围在13岁-82岁之间。从表 1可见只有1例（2.22%）有石棉接触史。职业主要为行政工作者和退休人员。

**1:** 45例MPM患者的临床特征 The clinical characteristics of the 45 patients with MPM

Characteristics	*n*	Proportion (%)
Gender (male)	30	66.67
History of asbestos exposure (Yes)	1	2.22
Smoking (Yes)	18	40
Occupation		
Cadres	18	40
Retiree	8	17.78
Farmer	5	11.11
Teacher	4	8.89
Student	2	4.44
Worker	1	2.22
Others	2	4.44
MPM: malignant pleural mesothelioma.		

### 2.2 临床表现

MPM的主要临床表现为胸痛、胸闷气促、咳嗽（表 2）。

**2:** 45例MPM患者的主要症状 The main symptoms of the 45 patients with MPM

Symptoms	*n*	Proportion (%)
Chest pain	24	53.33
Chest distress	22	48.89
Cough	17	37.78
Fever	15	33.33
Painful shoulder and back	6	13.33
Epigastric discomfort	5	11.11
No symptoms	5	11.11
Expectoration	3	6.67
Night sweat	1	2.22

### 2.3 CT表现

MPM的CT扫描结果主要表现为胸膜增厚、胸腔积液、肺部阴影及胸膜结节（表 3）。

**3:** 45例MPM的CT扫描结果 The CT scan of the 45 patients with MPM

CT Scan	*n*	Proportion (%)
Pleural thickening	32	71.11
Pleural effusion	27	60.00
Lung shadow	18	40.00
Pleural nodules	16	35.56
Tumor invasion ribs	6	13.33
Metastatic lymph nodes of mediastinum	6	13.33
Pleura mass	5	11.11
Encapsulated effusion	4	8.89

### 2.4 实验室检查

45例MPM患者中有27例患者合并胸腔积液，入院后行胸腔穿刺送检：其中，15例（55.56%）为黄色胸液，12例（44.44%）为血性胸液。胸水细胞学检查见恶性间皮细胞仅2例（7.41%）。胸水的常规和生化指标见表 4。从表 4可以看出，27例患者的胸水性质都为渗出液，且有核细胞数明显增多，以单核细胞的增多为主，乳酸脱氢酶明显增高，腺苷脱胺酶维持在正常范围。

**4:** 27例MPM患者的胸水检测结果 The detection for pleural effusion of the 27 patients with MPM

Index	Average (range)
Specific gravity	1.022 (1.016-1.027)
The total of nucleated cells (^*^10^6^/L)	5, 570 (1, 600-12, 060)
The total of white blood cells (^*^10^6^/L)	1, 520 (380-5, 130)
The proportion of multinucleate cells	0.23 (0.05-0.38)
The proportion of mononucleate cells	0.84 (0.62-0.95)
The total protein (g/L)	40.1 (35.6-53.3)
Lactate dehydrogenase (U/L)	405.3 (180.5-992)
Adenosine deaminase (U/L)	14.9 (5.8-25)

### 2.5 45例恶性胸膜间皮瘤的分期

采用UICC推荐的MPM肿瘤分期法对45例MPM患者进行分期^[2]^，结果见表 5。

**5:** 45例MPM的分期结果 The staging of the MPM in 45 patients

TNM stage	The details of TNM	*n*	Total
Ⅰ	T1aN0M0	2	3
	T1bN0M0	1	
Ⅱ	T2N0M0	6	6
Ⅲ	T1N1M0	1	10
	T1N2M0	1	
	T2N1M0	2	
	T3N0M0	3	
	T3N1M0	2	
	T3N2M0	1	
Ⅳ	T3N3M0	2	14
	T4N3M0	2	
	T3N3M1	1	
	T4N1M1	5	
	T4N3M1	4	
Unknown		12	12

### 2.6 确诊方法及病理组织细胞学检查

经胸腔镜检查后取组织18例（40%），开胸手术活检11例（24.4%），CT引导下经皮穿刺活检9例（20%），B超引导下胸膜活检7例（15.6%）。34例患者的病理组织免疫组织化学检测结果见表 6。

**6:** 34例MPM患者的免疫组织化学标记结果 The results of immunohistochemistry of 34 patients with MPM

Style	Epithelial (*n*=16)			Fibrosarcoma (*n*=11)			Mixed cell type (*n*=7)	
	Positive	Rate (%)		Positive	Rate (%)		Positive	Rate (%)
Calretinin	14	87.50		10	90.91		6	85.71
CK5/6	11	68.75		4	36.36		5	71.43
EMA	12	75.00		2	18.18		6	85.71
Podoplanin	9	64.29		3	27.27		5	71.43
CEA	1	6.25		0	0		1	14.29
P63	0	0		0	0		0	0
CD34	0	0		1	9.09		0	0
TTF-1	0	0		0	0		1	0
Vimentin	14	87.50		11	100		7	100
CK5/6: cytokeratin 5/6; EMA: epithelial membrane antigen; CEA: carcinoembryonic antigen; TTF-1: thyroid transcription factor-1.								

### 2.7 误诊疾病

45例患者中有39例患者由于主要症状无特异性被误诊，误诊率高达86.67%。误诊的疾病主要为结核性胸膜炎、肺癌伴胸膜转移，肺炎等（表 7）。

**7:** 39例MPM的误诊疾病 The misdiagnosis diseases of the 45 patients with MPM

Misdiagnosis diseases	*n*	Proportion (%)
Tuberculous pleurisy	13	33.33
Pleura metastasis of lung cancer	7	17.95
Pneumonia	6	15.38
Effusion caused by Pneumonia	6	15.38
Mediastinal tumor	3	7.69
Chronic gastritis	3	7.69
Tumor of ribs	1	2.70

### 2.8 治疗

在45例患者中，治疗方式为单纯全身化疗的病例数最多，所采用的化疗方案为顺铂联合培美曲塞（10例）、顺铂联合紫杉醇（3例）、顺铂联合吉西他滨（2例）、卡铂联合培美曲塞（1例）。疗效评估依据改良的RECIST标准^[3, 4]^：通过CT测量病灶与胸壁垂直的最短直径，选择3个独立水平，每个水平2个病灶，且每个水平相距至少1 cm。完全缓解（complete response, CR）：所有目标病灶消失，无新病灶出现，并维持4周；部分缓解（partial response, PR）：所有（1个或多个）基线目标病灶最长径总和减少30%，并维持4周；进展（progressive disease, PD）：较已记录的最小目标病灶最长径总和增大20%，或出现1个或多个新病灶。稳定（stable disease, SD）：所有基线目标病灶总和缩小但未达到PR，或增大但未达PD。疾病控制率（disease control rate, DCR）为CR、PR、SD之和除以总人数。从表 8可见，晚期的MPM（Ⅲ期、Ⅳ期）患者多采用化疗，而早期的患者（Ⅰ期、Ⅱ期）多采用根治性或姑息性手术治疗。45例患者中，疗效为PD的有21例（46.67%）。本文16例化疗患者中，有5例达到疾病控制，DCR为31.25%。根治性手术加放疗或化疗的患者中有2例疗效评估为CR，DCR为38.46%（表 8）。表 9显示，分期为Ⅰ期和Ⅱ期的9例MPM患者中，疗效评估为疾病控制有5例，DCR为55.56%，而分期为Ⅳ期的14例患者中，疗效评估为疾病控制仅有2例，DCR为14.29%。16例病理类型为上皮型的患者中，DCR为37.5%，而11例病理类型为肉瘤型的患者中，DCR为18.18%。27例有胸水的患者中，DCR为18.52%，14例无胸水的患者中，DCR为42.86%。

**8:** 45例MPM患者的不同的治疗方式与分期和疗效情况 Different treatments of the 45 patients with MPM according to the staging and efficacy

Treatment	Stage						Efficacy				
	Ⅰ	Ⅱ	Ⅲ	Ⅳ	Unknown		CR	PR	SD	PD	Unknown
System chemotherapy	0	0	4	10	2		0	2	3	9	2
Intrathoracic instillation and system chemotherapy	0	0	3	1	0		0	0	1	3	0
Radical surgery and chemotherapy/radiotherapy	3	3	0	0	0		2	0	1	3	0
Palliative surgery and chemotherapy/radiotherapy	0	3	3	0	1		0	1	1	4	1
Supportive treatment	0	0	0	3	1		0	0	0	3	1
Unknown	0	0	0	0	8		0	0	0	0	8
CR: complete response; PR: partial response; PD: progressive disease; SD: stable disease.											

**9:** 不同的治疗效果与分期，病理和胸水的关系 Different efficacy according to the staging, pathology and pleural effusion

Efficacy	Staging						Pathology					Pleural effusion		
	Ⅰ	Ⅱ	Ⅲ	Ⅳ	U		E	F	M	U		Yes	No	U
CR	2	0	0	0	0		2	0	0	0		0	2	0
PR	0	1	2	0	0		1	1	1	0		1	2	0
SD	0	2	2	2	0		3	1	1	1		4	2	0
PD	1	2	6	11	2		9	8	4	1		16	6	0
U	0	1	0	1	10		1	1	1	9		6	2	4
U: unknown; E: epithelial; F: fibrosarcoma.														

## 3 讨论

MPM是原发于胸膜的肿瘤，在欧洲其发病率为20/百万，占恶性肿瘤的0.02%-0.40%，其发病近年有增多趋势^[5]^。国外多数学者认为其发病与石棉有关，但本研究中45例患者仅1例有石棉接触史，考虑可能与其他致病原因如猿猴病毒40（SV40）的感染、非石棉化学纤维物质的接触和辐射等有关，亦或与东西方人遗传素质不同有关。

该病起病隐匿，临床表现缺乏特异性，主要表现为胸痛、胸闷气促和咳嗽，并可伴有发热和肩背痛^[6]^，与本研究结果一致。胸痛为持续性，且不随积液增多而减轻，一般镇痛剂难以缓解。影像学检查在MPM的诊断中占有重要的地位，特别是CT扫描常可发现常规胸片难以发现的胸膜病变，如胸膜弥漫性增厚，胸膜多发性结节。但当MPM转移到肺时，CT很难区分原发胸膜肿瘤与肺癌伴胸膜转移^[6, 7]^。

本研究中27例（60%）患者出现胸腔积液，与国外报道的60%-90%相一致^[6]^。相关文献^[1, 6]^报道MPM的患者多为血性胸液，而本组病例黄色胸液超过大半，因此不能根据颜色来判定良恶性，因为随着病程的进展，MPM患者的胸水可由原来的草黄色变为血性。胸腔积液检查的意义较大，当胸水中发现间皮细胞在5%以上提示可疑本病，本组27例胸水患者中发现间皮细胞仅有2例（7.41%）。目前不推荐单独根据胸水细胞学检查结果来诊断MPM，因为MPM的瘤细胞与增生活跃的间皮细胞及转移性腺癌细胞在光镜下常难以鉴别。胸水化验均显示为渗出液，细胞数以单核细胞增多为主，乳酸脱氢酶明显高于正常，而腺苷脱胺酶在正常范围，据此可与结核性胸膜炎及炎性积液相鉴别。

肿瘤的分期不仅能指导临床医生选择治疗方案，而且能较好地预测疾病的预后^[2]^。对于其它实体肿瘤，已经有一种能被大家接受的肿瘤分期方法。而在MPM领域至少有5个分期系统^[2]^，但没有一种被广泛接受。其最新的分期是由国际间皮瘤研究组织设计完成，并获得UICC认可。在影像学技术中，CT被认为是最有效的，且被广泛应用，但准确性仍有待提高。本研究中采用的是最新分期法，大部分的患者为Ⅲ期和Ⅳ期，大多发现时已经失去手术机会，普遍预后欠佳。

本组中40%的患者确诊的方式是通过胸腔镜，文献^[8]^报道胸腔镜可以为90%的病例提供确切的诊断。它可直接观察胸膜病变部位、形态、大小，同时可在直视下进行多点、足够深和足够大的活检，且比开胸活检安全性高。因此除有手术禁忌症或胸膜粘连的患者，都推荐行胸腔镜检查来诊断MPM。当CT显示有肺部病灶或胸膜肿块明显时，则可进行CT引导下肺穿刺活检或B超引导下胸膜穿刺活检进行明确诊断。

MPM按细胞学类型可分为上皮型、纤维肉瘤型及混合型3种，分别占50%、34%及16%。它作为一种从浆膜腔间皮细胞进展而来的恶性肿瘤，有多种不同的细胞异型性，易产生各种误导组织病理学诊断的陷阱，因而其确诊应基于免疫组化检查^[9]^。为鉴别上皮样间皮瘤和腺癌，指南推荐采取两种具有间皮瘤阳性诊断价值的标记物和两种具有阴性诊断价值的标记物联合。常用的具有阳性诊断价值的标记物为钙视网膜蛋白（Calretinin）、膜标记物抗上皮膜抗体（EMA）、波形蛋白（Vimentin）、抗D2-40抗体（podoplanin）和抗细胞角蛋白抗体（CK5/6），常用的具有阴性诊断价值的标记物为CEA和TTF-1^[9]^。其中CEA几乎对腺癌有100%的特殊性和敏感性^[10]^。为区分肉瘤样间皮瘤与鳞癌，指南推荐使用2种广谱的抗角蛋白抗体和两种具有阴性预测价值的标记物（如抗CD34抗体和抗S100抗体）等。本研究中上皮型中钙视网膜蛋白（Calretinin）、膜标记物抗上皮膜抗体（EMA）和抗细胞角蛋白抗体（CK5/6）阳性率在70%左右，而仅1例CEA阳性。而在肉瘤型中波形蛋白（Vimentin）和钙视网膜蛋白（Calretinin）阳性率较高，CEA全部为阴性。

MPM生存期短，预后极差，现有的治疗手段包括：化疗、胸腔内灌注化疗、根治性手术、姑息性手术和支持治疗，但这些只能有限的延长生存期或改善生活质量^[9]^。目前，顺铂+培美曲塞已被美国食品与药品管理局和欧洲医药评价署批准为治疗MPM的一线标准化疗方案^[11]^。根治性手术是通过胸膜外肺切除术切除整个胸膜、肺、心包膈膜以及系统结节清扫。姑息性手术是指胸膜部分切除术或剥除术，可去除壁层肿瘤组织缓解限制性通气不足和胸壁痛，去除脏层胸膜解除压迫导致的肺不张。但单独的外科手术不能治愈，因为胸膜内层，特别是在心包膜和纵隔边缘的1 cm-2 cm不能被切除，所有的外科手术切缘都是阳性。Schipper等^[12]^报道术后辅助化疗能有效的降低MPM的病死率。本调查中早期的患者多以手术的患者为主，而晚期的患者则以化疗和对症治疗如止痛、镇咳、缓解呼吸困难等为主。疗效的评估可以看出，大多非手术病人为SD和PD，很少有达到CR和PR的。而早期手术的病人，可能达到CR。本调查中，病理类型为上皮型的DCR要高于肉瘤型，无胸水患者的DCR要高于有胸水的患者，而晚期患者DCR要低于早期的患者。Sugarbaker等^[13]^曾报道病理类型是影响MPM预后的主要因素。上皮型MPM对治疗反应和预后均明显好于非上皮型。曾提出如同小细胞肺癌和非小细胞肺癌一样，应该将上皮型和非上皮型MPM分别进行研究。目前此观点尚未达成一致。

总之，MPM缺乏特征性表现，尤其是早期诊断十分困难，误诊率极高，在临床上遇到下列情况应怀疑MPM：①肺内无明显病变的胸腔积液伴发持续性胸痛的患者；②影像学提示有胸膜增厚或胸膜结节的患者；③胸水常规生化提示恶性积液，且细胞学找到肿瘤细胞的患者；④抗结核治疗效果不佳的结核性胸膜炎患者。可进一步行胸腔镜或开胸肺活检取病理组织，结合免疫组化的结果尽早确诊。该病恶性程度高，目前无论单一治疗手段还是综合治疗都不能取得满意效果。
